# Whole Genome Sequencing of Newly Established Pancreatic Cancer Lines Identifies Novel Somatic Mutation (*c*.*2587G>A*) in Axon Guidance Receptor Plexin A1 as Enhancer of Proliferation and Invasion

**DOI:** 10.1371/journal.pone.0149833

**Published:** 2016-03-10

**Authors:** Rebecca Sorber, Yaroslav Teper, Abisola Abisoye-Ogunniyan, Joshua J. Waterfall, Sean Davis, J. Keith Killian, Marbin Pineda, Satyajit Ray, Matt R. McCord, Holger Pflicke, Sandra Sczerba Burkett, Paul S. Meltzer, Udo Rudloff

**Affiliations:** 1 Thoracic & GI Oncology Branch, Center for Cancer Research, National Cancer Institute, NIH, Bethesda, Maryland 20892, United States of America; 2 Genetics Branch, National Cancer Institute, NIH, Bethesda, Maryland 20892, United States of America; 3 Surgery Branch, Center for Cancer Research, National Cancer Institute, NIH, Bethesda, Maryland 20892, United States of America; 4 Molecular Cytogenetic Section, MCGP, Center for Cancer Research, National Cancer Institute, NIH, Frederick, Maryland 21702, United States of America; 5 Department of Biology and Center for Cancer Research, Tuskegee University, Tuskegee, Alabama 36088, United States of America; Vrije Universiteit Brussel, BELGIUM

## Abstract

The genetic profile of human pancreatic cancers harbors considerable heterogeneity, which suggests a possible explanation for the pronounced inefficacy of single therapies in this disease. This observation has led to a belief that custom therapies based on individual tumor profiles are necessary to more effectively treat pancreatic cancer. It has recently been discovered that axon guidance genes are affected by somatic structural variants in up to 25% of human pancreatic cancers. Thus far, however, some of these mutations have only been correlated to survival probability and no function has been assigned to these observed axon guidance gene mutations in pancreatic cancer. In this study we established three novel pancreatic cancer cell lines and performed whole genome sequencing to discover novel mutations in axon guidance genes that may contribute to the cancer phenotype of these cells. We discovered, among other novel somatic variants in axon guidance pathway genes, a novel mutation in the PLXNA1 receptor (c.2587G>A) in newly established cell line SB.06 that mediates oncogenic cues of increased invasion and proliferation in SB.06 cells and increased invasion in 293T cells upon stimulation with the receptor’s natural ligand semaphorin 3A compared to wild type PLXNA1 cells. Mutant PLXNA1 signaling was associated with increased Rho-GTPase and p42/p44 MAPK signaling activity and cytoskeletal expansion, but not changes in E-cadherin, vimentin, or metalloproteinase 9 expression levels. Pharmacologic inhibition of the Rho-GTPase family member CDC42 selectively abrogated PLXNA1 c.2587G>A-mediated increased invasion. These findings provide *in-vitro* confirmation that somatic mutations in axon guidance genes can provide oncogenic gain-of-function signals and may contribute to pancreatic cancer progression.

## Introduction

Pancreatic cancer remains a fatal condition. The 5-year survival rate of patients afflicted by the disease of less than 5 percent has not changed over the last three decades [1]. One of the main reasons for this lack of progress is the inability to provide patients with more effective treatment options [2, 3]. For example, erlotinib, in combination with gemcitabine, received regulatory approval as the first molecular therapy in advanced pancreas cancer based on both a progression-free and overall survival difference of slightly more than two weeks between the gemcitabine plus erlotinib group and patients having received gemcitabine only [4]. While there has been recently regulatory approval of the chemotherapy triplet (FOLFIRINOX) and the combination of gemcitabine and nab-paclitaxel (Abraxane^®^) improving outcome from 6.8 and 6.7 months in the gemcitabine-only control arm to 11.1 and 8.5 months, respectively, there have been no breakthroughs in the molecular therapy arena for patients with pancreatic cancer so far [2, 5, 6].

One of the strategies to accelerate progress has been the deployment of improved deep sequencing technologies to interrogate pancreatic cancer genomes for novel somatic variants in genes, or signaling pathways, which can be exploited as targets for personalized molecular therapy efforts. While initial results of the recently released ‘Individualized Molecular Pancreatic Cancer Therapy (IMPaCT) Trial’ designed to exploit results from genome sequencing of pancreatic cancer highlighted some of the challenges of the genotype-directed molecular therapy approach, it is expected that the ongoing evolvement and improvement towards miniaturization, automation, and clinical applicability together with decreasing costs will bring both rare and novel variants into the arena of clinically valuable targets [7, 8]. One such novel signaling network found to be affected by a large number of genetic perturbations within a large deep-sequencing effort is the axon guidance (AG) signaling pathway [9, 10]. Axon guidance is a process that is well described in neuronal development and illustrates how cues from the microenvironment guide axons to specific locations where synapses are formed. These genes are highly expressed in GI cancers, and a couple of studies have suggested that dysregulation of these genes aid in the progression of pancreatic cancer. For instance, whole genome sequencing of 99 pancreatic tumors identified structural variants of AG genes in up to 25 percent of cancer genomes. These genes include SLIT ligands and their ROBO receptors, or the semaphorin ligands (SEMA) and their plexin receptors (PLXN) [9]. Expression levels of AG genes strongly correlate with clinical outcome of pancreas cancer patients. Low expression levels of ROBO2 receptors and SLIT ligands, affected in ≥10% by genomic deletions in pancreas cancer, were found more frequently in patients with poor outcome suggesting a tumor suppressing role. High expression levels of the PLXNA1 receptor and semaphorin 3A (SEMA3A) ligand on the other hand were measured in patients with shorter survival suggest a gain-of-function of these AGs in pancreas cancer. Other studies linking genetic alterations of AG genes to a tumor promoting pro-survival role in pancreas cancer are a recent mutagenic transposon screen in mice, the detection of AG gene mutations in early precancerous lesions, genome wide association studies suggest genome-wide methylation patterns in pancreas cancer with recurrent perturbations in AG genes, or other correlative gene and protein expression studies in cancer specimens and clinical outcomes [9, 11–14]. While three sequencing efforts independently validated the presence of structural variants in AG genes and a recent study showed that the SLIT2-ROBO2 axis functions as a tumor suppressor in pancreas cancer, there have been no *in vitro* studies interrogating the impact of somatic AG gene mutations on pancreas cancer biology [9, 15–17].

In this study, we established three new pancreatic cell lines and discovered, among other somatic AG mutations, a novel gain-of-function mutation in a plexin (PLXNA1) receptor in one of these lines. Activation of the mutated, but not wild type, receptor via its natural ligand SEMA3A dramatically increased invasive and proliferative properties. Pancreatic cancer cells expressing mutant PLXNA1 showed activation of Rho-GTPase CDC42, cofilin, and MAPK signaling which was accompanied by cytoskeletal and expansion disorganization. Increased invasion in mutant PLXNA1 cells returned to baseline upon CDC42 inhibition without affecting wild type PLXNA1 expressing cells. We suggest that somatic variants in AG genes have a pro-oncogenic function in epithelial cancer cell lines.

## Methods

### Patients and Protocols

This study received ethical approval from the National Institutes of Health/National Cancer Institute Institutional Review Board (Bethesda, MD) under protocol NCI-09-C-0079, "The Natural History of Solid Organ Cancer Stem Cells (SOCSC)". Informed, written consent was obtained from all patients for surgical procedures, as well as use of samples for correlative tissue studies including establishment of cell lines and cancer genetic studies. Only patients with a histologically confirmed diagnosis of cancer were enrolled. All patients were treated at the Center for Cancer Research (CCR), National Cancer Institute, Bethesda, MD, in accordance with the study principles expressed in the Declaration of Helsinki [18].

### Cell culture of tumor specimens

Cell lines were established from three patients with pancreatic ductal adenocarcinoma, one with disease confined to the pancreas and two from patients whose metastases from their pancreatic primary were sampled (ileal lymph node and liver metastasis). All surgical specimens (taken from the peripheral viable and glistening portion of tumor) were transported in 4°C Dulbecco's Modified Eagle Medium (DMEM; Gibco, Grand Island, NY) and immediately processed in the laboratory. Remaining uninvolved pancreatic tissue and soft tissue was dissected off and tumors were minced into cubes of 1- to 3-mm. Cell lines were established using two methods, either tumor outgrowth followed by direct culture in specialized pancreatic cancer cell growth media, or by using spheroid cultures[19–21]. In brief, cell lines established by outgrowth and direct culture were derived from 1mm^3^ minced tumor fragments cultured for 6 to 16 weeks in initial primary pancreatic cancer cell growth media containing 20% fetal bovine serum (FBS, Hyclone, Logan, UT), Roswell Park Memorial Institute (RPMI) 1640 (Lonza) supplemented with 200μM L-glutamine (Invitrogen, USA), 100 units/ml penicillin/streptomycin (Invitrogen, USA), 1% sodium pyruvate (Invitrogen, USA) and non-essential amino acids (Invitrogen, USA). The media was also supplemented with human insulin-like growth factor 1 and 2 (10μg/ml each, Sigma, USA), and 0.2 units/mL human insulin (Sigma, USA). Media was changed every 3 to 4 days. Stromal cells, cellular debris, and remaining non-cancer cells were removed by controlled trypsinization after growing pancreas cancer cell islands and expanded colonies were observed. Cultures were transitioned to 5% FBS RPMI 1640 with above supplements with the exception of insulin and insulin-like growth factors. Cell line SB.12 followed a protocol previously described for the establishment of colon cancer lines [19]: in brief, tumor tissue was subject to a combination of mechanical and enzymatic digestion by gentleMACS C tube (Miltenyi Biotec, Germany) and dissociation medium containing DMEM/F12 (1:1) growth medium (Mediatech, Herndon, VA., USA). A short-term primary organoid culture was established on ultra-low attachment T-25 flasks (Corning, Tewksbury, MA, USA) using DMEM/F12 (1:1) and grown for 7 days. Spheroid-bodies were harvested from primary fresh tumor suspension cultures, and injected subcutaneously into SCID mice. Tumors were grown to 2cm3, explanted, re-digested, and cultured as described above on normal adherent tissue culture flasks.

All adherent cultures were passaged at confluence and media was changed every 3–4 days. All cells were maintained at 37°C in humidified air and 5% CO_2_. Cell lines were not grown beyond passage 5 to 8 and tested free of mycoplasma.

### Cell culture growth in vitro

Cell lines Panc1, Panc01.28, Panc06.05, Panc08.13, and Panc10.05 were a kind gift of Dr. Elizabeth Jaffe (Johns Hopkins Sidney Kimmel Comprehensive Cancer Center) and were grown as previously described [20]. Doubling time was determined by seeding 3×104 cells in triplicates in 6-well dishes and counting viable cells in triplicate for each well (trypan blue dye exclusion method using a T4 Cellometer (Nexcelom Bioscience, Lawrence, MA)) every 24 hours. The doubling time (DT) was calculated from the logarithmic portion of the growth curve using the formula DT = T × ln2 / ln(Xe/Xb) with T, incubation time in any units; Xb, cell number at beginning of incubation time; Xe, cell number at end of incubation time.

### Nucleic acid extraction from cell lines and matched blood, HLA typing

Genomic DNA and total RNA from washed cell pellets, matched blood (SB.06, SB.12) and matched buffy coat (SB.07) were isolated with DNeasy and RNeasy kits (Qiagen, Valencia, CA), extraction of genomic DNA from liver tissue was aided with the QIAshredder system (Qiagen, Valencia, CA). HLA sequence analysis facility at NCI Immunogenetics Laboratory was utilized for defining the genotypic identity of HLA alleles of both classes of HLA genes, class I (including HLA-A, HLA-B and HLA-C), and class II (HLA-DRB1, DRB3, DRB4, DRB5, DPB1, DPA1, DQB1, and DQA1).

### Cytogenetics—spectral karyotyping (SKY)

Slides for SKY were prepared from cells arrested in metaphase after treatment with 10ng/ml vinblastine for 4 hours. Human SKY probes (Applied Spectral Imaging (ASI), Carlsbad, CA, USA) of flow cytometry-sorted human chromosomes labeled with different fluorochromes were used. Denaturation, hybridization, and detection procedures were performed according to the manufacturer's protocol. Chromosomes were counterstained with 4', 6-diamidino-2-phenylindole (DAPI). Spectral images were acquired with the SD200 Spectracube system (ASI) attached to a Nikon Eclipse 800 microscope (Nikon, Melville, NY, USA) with a single exposure of the image by using a custom-designed triple-band pass filter (Chroma Technology, Brattleboro, VT, USA). An interferometer measured the spectrum at each pixel of the image. A discrete color was assigned to all pixels with identical spectra according to a spectrum classification system, and the pixels were then displayed in the assigned colors. Five to eight metaphase chromosome spreads from each sample were analyzed by SKY.

### Histological and morphological evaluation of tumors

Tumor tissues were fixed in 4% paraformaldehyde, embedded into paraffin blocks, and 6 micron sections were fixed onto glass microscope slides. All cellular morphology and histology was determined by phase-contrast microscopy (Olympus IX51, Zeiss) by a board-certified pathologist.

### Immunohistochemistry

Carcinoembryonic antigen (CEA) rabbit polyclonal anti-human CEA primary antibody (1:4,000, DAKO Glostrup, Denmark, Cat#A0115) or and biotinylated goat anti rabbit IgG (1:100, Vector Labs, Burlingame, VT, USA), mouse monoclonal anti human CA19-9 primary antibody (1:40, AbD Serotec USA, Cat #MCA1913T), and secondary antibody were applied for 30 minutes at room temperature to mounted 6 micron tumor sections. Staining was performed using ABC Elite (Vector Labs, USA). DAB was used for 5 min and hematoxylin was used as counterstain. Staining was performed using Mouse on Mouse Polymer HRP kit of MaxVision, Biosciences Inc, USA. DAB was used for 5 min and hematoxylin was used as counterstain.

### Immunofluorescence

50,000 cells were centrifuged onto charged glass slides and fixed with methanol at room temperature for 2 min. Cells were permeabilized in PBS containing 0.25% Triton-X-100 for 10 minutes at room temperature. Mouse monoclonal anti human CA19-9 primary antibody (2 μg/ml, AbD Serotec USA, Cat # MCA1913T) and isotype control (2μg/ml, AbD Serotec USA, Cat # 0200–0473) were used. Mouse monoclonal anti human CEA primary antibody (2μg/ml, Cell Signaling USA, Cat # 2383) with isotype control (2μg/ml, Cell Signaling USA, Cat # 5415) and E-cadherin, mouse monoclonal anti-E-Cadherin primary antibody (2μg/ml, BD Biosciences, USA) with isotype control (2μg/ml, BD Biosciences, USA) were used. Alexa Fluor^®^ 488 goat anti-mouse IgG (H+L) (5μg/ml, Life Technologies, USA) was used as secondary antibody. Alexa Fluor^®^ 555 Phalloidin (Cell Signaling USA, Cat # 8953) was used to stain the cytoskeleton. Slides were mounted with Vectashield/DAPI stain (Vector Laboratories H-1200) and stored at 4°C in darkness. Images were captured with Olympus IX51 microscope with UV light source and appropriate filters. A 20x objective was used for DAPI (blue) and Alexa-Fluor 488 (green). Images were merged with software of the same microscope.

### Whole genome sequencing and variant calling

Sequencing of the cancer genomes of SB.06 and SB.07 and matched normal was performed on the Complete Genomics platform (Complete Genomics, Mountain View, CA) [7, 9]. Reads were aligned to the NCBI build 37 reference genome. Variations between tumor and matched normal were called using the software tool CGA Tools^™^ which included algorithms for somatic single nucleotide variants and indels. The calldiff function of cgatools (http://cgatools.sourceforge.net) assigns a somatic score to each SNV and to short deletions and insertions as a measure that each SNV or indel is truly a somatic variant [7]. Exomic variants have been deposited in dbGaP (ID# phs001042.v1.p; at http://www.ncbi.nlm.nih.gov/projects/gap/cgi-bin/study.cgi?study_id=phs001042.v1.p1).

### Targeted cancer gene assay

DNA (500ng) was fragmented using a Covaris S2 Focused ultrasonicator to a mean size of 300 bp. Fragment ends were repaired and phosphorylated with T4 DNA polymerase (New England Biolabs, USA), Klenow fragment (New England Biolabs, USA), and T4 polynucleotide kinase (New England Biolabs, USA). A 3’ A-base overhang was introduced with Klenow exo-minus (New England Biolabs, USA) and fragments were ligated to Illumina paired-end adaptors. Ligation products with a mean size of 350 bp +/- 20% were isolated on a Caliper LabChip XT (PerkinElmer) and amplification was performed using Illumina PCR primers InPE1.0 and InPE2.0 and primer indices. Pooled, indexed libraries were captured using an Agilent SureSelect Custom DNA kit targeting exons of 197 commonly mutated cancer genes (Agilent Technologies, USA) according to the manufacturer’s protocol and sequenced at a depth of 67x and 163x for SB.07 and SB.06, respectively. Alignments to the hg19 human reference genome assembly were performed with BWA version 0.6.2-r126 [22] and duplicates were marked with picard [23]. Single nucleotide variants were called with the samtools package [23] and small insertions and deletions were called with pindel version 0.2.5 [24].

### Sequence confirmation

In order to confirm expression of PLXNA1 WT and c.2587G>A mutants in transfected 293T cells a 361 bp amplicon was generated by PCR from cDNA made from transfected cells. Custom primers *AAGGCCGACCCGCGCTTCGA* and *ACACACCTCCACCAGGGAGT* were purchased from Invitrogen (Carlsbad, CA, USA). Standard Sanger’s sequencing analysis was used to generate and analyze the amplicon for mutations.

For confirmation of the PLXNA1 variant in original tissues, the Life Technologies Taqman Custom Assays (Life Technogies, Grand Island, NY) was used. Assay was conducted with the forward primer GCGTCACGGCAGCAG and reverse primer GGGCCCTACCTTGAGGATC, with wild type PLXNA1 allele sequence CTGCACCGACCCCAA detected with VIC dye and mutant allele CTGCACCAACCCCAA with FAM dye. Concentrations of reference allele to mutant allele were calculated using software and instructions of the manufacturer.

### siRNA transfections and reverse transcription-PCR

siRNA for depletion of PLXNA1 was purchased from Qiagen (Cat# SI00142562, Valencia, CA). Efficacy of siRNA-mediated knockdown of the target gene was evaluated on the mRNA level by RT-PCR for each sequence in cell lines SB.06, SB.07, SB.12, Panc05.04, Panc08.13, and Panc10.05 following the manufacturer’s instructions. Total RNA from cells transfected was extracted with the RNAeasy kit (Qiagen, Valencia, CA). A total of 450ng of total RNA was used for single-strand complementary DNA synthesis using a SuperScript III First-Strand kit (Invitrogen, Carlsbad, CA). Complementary DNA was amplified using the oligo dT20 (Invitrogen-Life Technologies) primer supplied in the kit. To test for loss of target gene mRNA, we used 1μl of complementary DNA in the PCR reaction with target gene primers recommended by the manufacturer (Qiagen, USA).

### Cell proliferation assay of siRNA transfected cells

Cells were seeded at a density of 5×104 in 6-well plates and transfected with two different siRNAs yielding the most efficient knockdown as determined by RT-PCR. After 48 hours, cells were trypsinized, and re-seeded at a density of 2×103 per well in 96-well plates. Cell proliferation was determined at 24, 48, and 72 hours using the CellTiterGlo assay (Promega, Madison, WI) according to the manufacturer’s protocol.

### Construction of wild-type and mutant PLXNA1 expression vectors

Human PLXNA1 (NM_032242) were cloned from an ORF library into pENTER purchased from ViGene (ViGene, Rockville, MD) into the Multiple cloning site region of the plasmid. The c.2587G>A mutation was created by site-directed mutagenesis using Phusion PCR (New England Biolabs, Ipswich, USA) per manufacturer’s instructions. The site-directed mutagenesis primers used were 5’-*ACGGCAGCAGTCGCTGCAACCAACCCCAAGATCCTCAAGCT-3’* and 5’-*CAGCTTGAGGATCTTGGGGTTGGTTGCAGCGACTGCTGCCGT*-3’. Introduction of mutation into the plasmid was confirmed by sequencing.

### Transient expression and proliferation and invasion assays

293T cells were purchased from ATCC and transfected with GeneCarrierI (Epoch Life Science, Sugar Land, TX, USA) at a 4:1 (μl/μg) ratio with DNA using 3 to 5μg of plasmid DNA. To examine growth potential, 48 hours after transfection, cells transfected with empty vector, wild-type, or mutant PLXNA1 gene were seeded into 96-well plates at 2,500 cells per well and incubated for 10 days. Samples were analyzed every 48 hours by CellTiterGlo measurements according to manufacturer’s instructions (Promega, USA). Invasion assay was performed after 48 hours of transfection in 6-well plates using Boyden migration chambers (8 μm pore size; 12-well plate format; BD Biosciences, Palo Alto, CA) according to the manufacturer’s instructions. 5×104 transfected cells in 1mL DMEM with 10% FBS were seeded in duplicate into the upper chambers and grown overnight in the presence of 150ng/ml of SEMA3A (Peprotech, USA). The non-invading cells were removed from the upper surface of the membrane with a cotton- tipped swab and cells on the lower surface of the membrane were stained with 1% toluidine blue (Sigma-Aldrich, USA).

### Measuring transfection efficiency via Western Blot analysis

Transfected 293T cells were lysed with Cell Signaling lysis buffer (Cat#40–040, Millipore, Bellerica, USA). Protein concentration was determined via BCA analysis kit (ThermoScientific, Waltham, USA). Approximately 50ug of protein was loaded into a 12% SDS/Polyacrylimide gel. Proteins were transferred to nitrocellulose blotting paper via the dry HEP-OWL1 system (ThermoScientific, Waltham, MA). Nitrocellulose blots were incubated with Anti-Flag M2 (Cat#2368S, Cell Signaling, Danvers, MA) and anti-Actin (Cat#4967, Cell Signalling, Danvers, USA) antibodies. Bands were visualized via the Odyssey luminescence scanner (Li-Cor, Lincoln, USA) according to manufacturer’s instructions.

### RT-PCR

RNA was isolated from 5×104 293T cells transfected with PLXNA1 wild type and c.2587G>A mutant using the RNAeasy 50 extraction kit (Qiagen, Valencia, CA). cDNA was generated by SuperScript III First-strand kit (Life Technologies, Carlsbad, CA). The Rho pathway RT^2^ Profiler^™^ PCR Array (Qiagen, Valencia, CA) was loaded with cDNA according to manufacturer’s instructions, and read in a BioRad CFX96 cycler. Gene expression was quantified using Taqman assay (Invitrogen) and analysis was performed in triplicates. Taqman assay IDs used included E-cadherin (Hs-01023894), MMP9 (Hs-00234579), vimentin (Hs-00185584) and GAPDH (Hs-02758991).

### Dose response curves

The effects of erlotinib and NVP-BEZ235 (Selleck, Houston, TX) on proliferation were tested by seeding 5,000 cells per well in 96-well plates and incubating them for 24 hours before addition of drug. Increasing concentrations of drug were added to each well in three replicates with DMSO as negative control. Plates were analyzed 72 hours after addition of drug using the Promega CellTiterGlo assay reagent (Promega, Madison, WI). Plates were read with GloMax^®^ 96 Microplate Luminometer (Promega, Madison, WI), and the data analyzed using SoftMax version 5 and GraphPad Prism version (La Jolla, CA).

## Results

### Establishment, authentication, and characterization of human pancreas carcinoma lines SB.06, SB.07, and SB.12

All cell lines were established from adenocarcinomas of the pancreas of either primary or metastatic lesions. Cell lines SB.06 and SB.07 were derived from surgical specimens by tumor cell outgrowth method in special media [20], cell line SB.12 from spheroid short-term cultures grown from the surgical specimen and passaged through immunocompromised mice [3]. Table 1 summarizes the patients’ clinicopathological characteristics including tumor grade, serum CEA and CA19-9 levels at time of operation, and HLA type.

**Table 1:** Clinicopathological features of pancreatic ductal adenocarcinoma patients.

Cell line	Gender	Age (yrs.)	1° site, pancreas	Site of harvest	Stage*	Pre-treatment	Histo-pathologic differentiation	Invasion	Serum CEA [μg/L]	Serum CA19-9 [U/mL]
**SB.06**	Male	70	Tail	Primary	IIA	Chemo-therapy, radiation	Moderate to poor	++ perineural	4.5	473
**SB.07**	Female	69	Body	Ileal lymph node	IV	-	Moderate to poor	-	16.7	174
**SB.12**	Male	70	Tail	Left liver	IV	Chemo-therapy	Poor	-	142	46,940

*7^th^ Edition, Pancreas Cancer Staging, American Joint Committee on Cancer, 2010.

**Fig 1:**
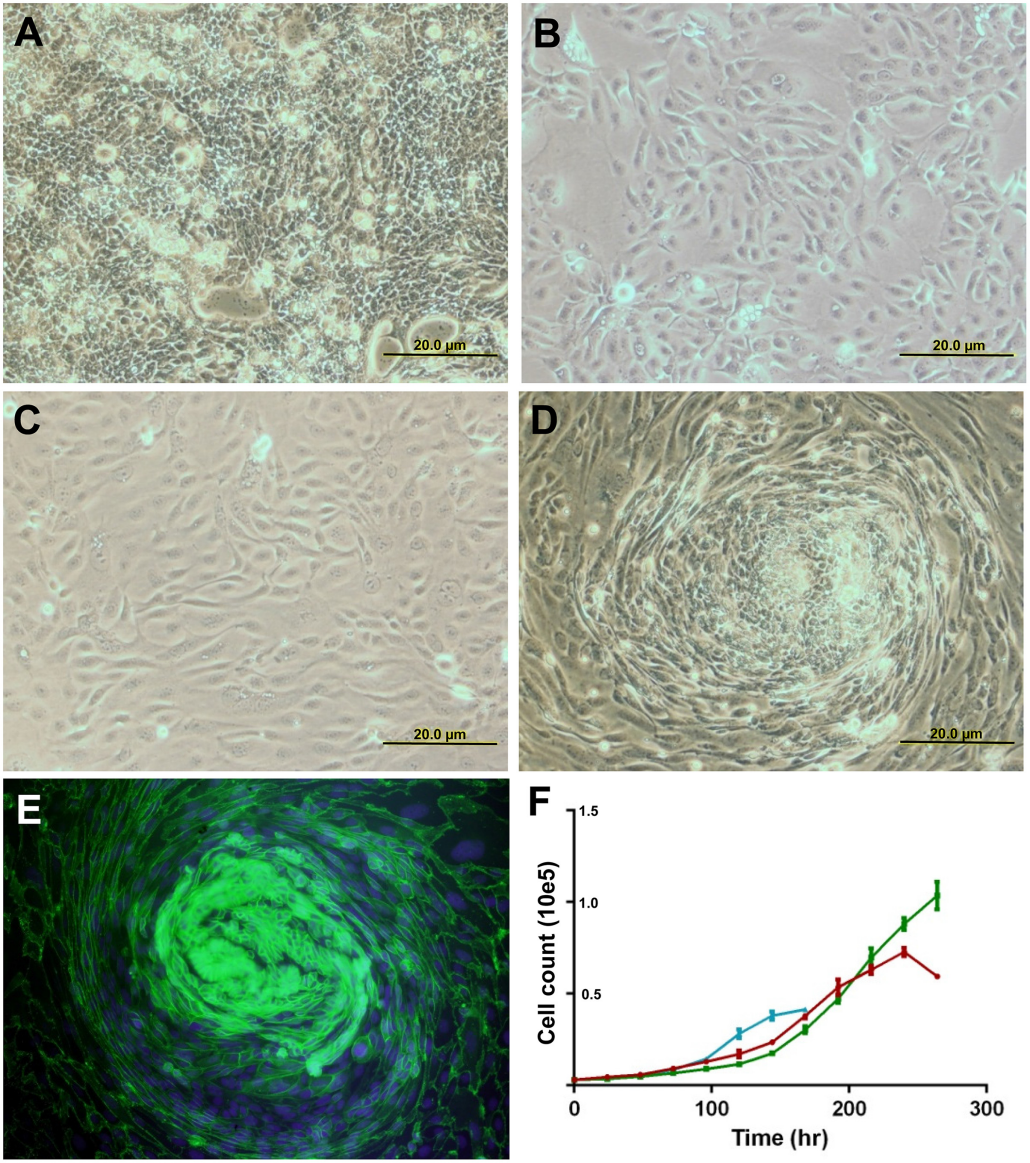
Growth characteristics of established cell lines. Phase contrast microscopy of cell lines A) SB.07, B) SB.12, and C) SB.06. Formation of a ‘swirl’-like growth pattern (D) in cell line SB.06 at subconfluence and with E-cadherin/DAPI immunofluorescent staining (E). (F) Growth curves for established cell lines SB.06 (red), SB.07 (green), and SB.12 (blue).

Single cells of each cell line were able to give raise to colonies and cell islands. There were two different morphologies: SB.07 cells predominantly grew in cohesive sheets and islands with close cell-cell contacts (Fig 1A). These cells were larger and round, and displayed limited heterogeneity. In contrast, SB.12 and SB.06 cells were smaller, spindly with atypical elongations, and displayed a non-contact growth pattern (Fig 1B and 1C). At subconfluence, SB.06 shows the development of a swirl-like pattern (Fig 1D and 1E). Growth curves of the cell lines are shown in Fig 1F, doubling times of SB.06, SB.07, and SB.12 are 45.9, 50.3, and 37.1 hours, respectively.

### Immunocytochemical profiling

**Fig 2:**
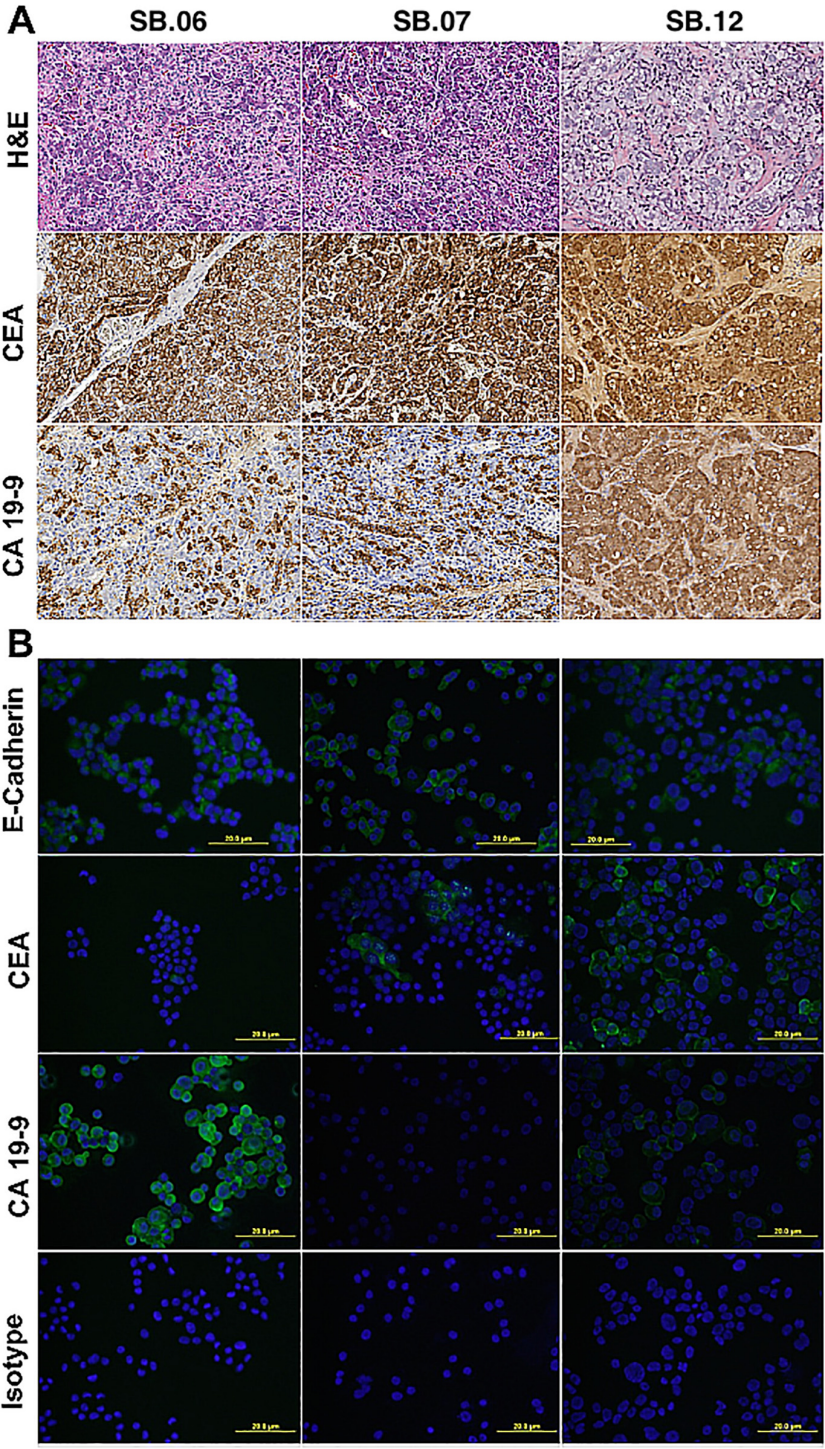
Immune phenotyping of parent tumor samples matches established cell lines. A) Immunohistochemical staining of parent tumor samples SB.06, SB.07 and SB.12 for CEA and CA-19-9; B) Immunofluorescence DAPI/Alexa-Fluor488 for E-cadherin, CEA, and CA19-9 of cell lines SB.06, SB.07, SB.12.

Cell lines derived from primary and metastatic lesions shared the immunohistochemical phenotype and HLA genotype with the corresponding primary tumors with the exception of CA19-9 expression in cell line SB.07. Primary SB.07 tumor showed weak CA19-9 positivity in less than 20 percent of cells while in cell line SB.07 CA19-9 expression was observed in less than 5 percent of cells. Fig 2 shows the immunohistochemical (A) and immunofluorescent (B) staining of tissues removed at surgery and derived cell lines, respectively, Table 2 summarizes results of phenotypic characterization and HLA typing of both primary tumor and cell lines. All cell lines grew tumors in mice, twice the number of SB.07 cells were required to grow tumors of same size of SB.06 within the same period of time (data not shown).

**Table 2:** Immunophenotypical characteristics of cell lines and corresponding parent tumors.

		Immune phenotype			HLA allelotype				
		*CEA*	*CA19-9*	*CDH1*	*A*	*B*	*C*	*DR*	*DQ*
**SB.06**	*Tumor*	**++**	**++**	**n/a**	01, 11	08, 55	03, 07	03, 1001	02, 05
	*Cell line*	**+**	**+++**	**+++**	01, 11	08, 55	03, 07	0301, 1001	02, 05
**SB.07**	*Tumor*	**++**	**++**	**n/a**	01, 0201	08, 44	05, 07	0301, 1301	0201, 06
	*Cell line*	**++**	**-**	**+++**	01	08	07	0301	0201
**SB.12**	*Tumor*	**+++**	**+++**	**n/a**	30, 32	08, 13	06, 07	---	---
	*Cell line*	**+++**	**++**	**++**	30, 32	08, 13	06, 07	---	---

### SKY karyotype analysis

**Fig 3:**
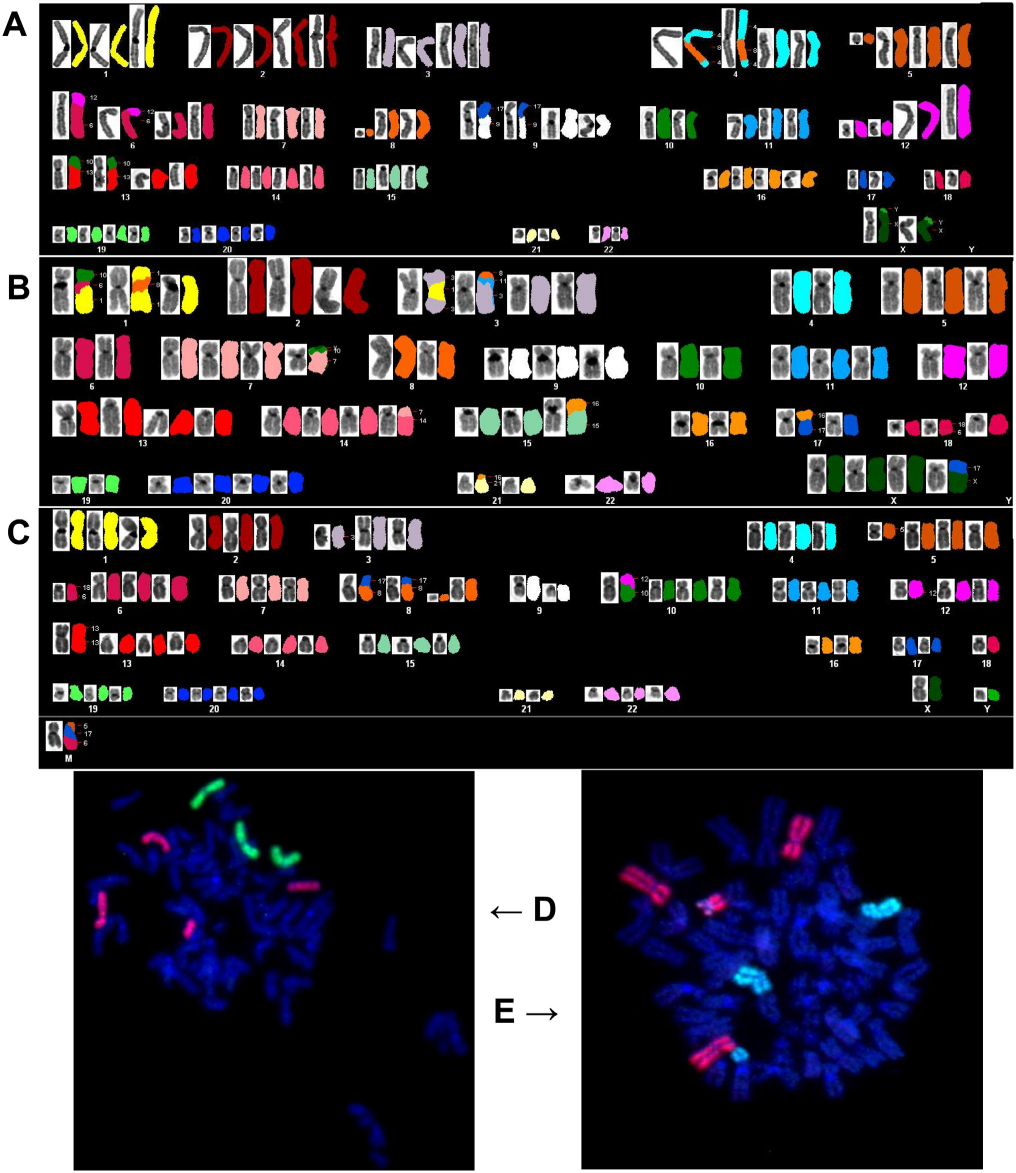
Chromosomal analysis of established cell lines. Karyograms of cell lines A) SB.06, B) SB.07, C) SB.12, and FISH validation of shown chromosomal translocations in cell lines D) SB.06, E) SB.07.

All cell lines were subjected to multicolor SKY karyotyping. All cell lines were diploid and had a human karyotype (Fig 3A, 3B and 3C). Several chromosomal translocations unique to each cell line were observed and validated with FISH (Fig 3D and 3E). The main observed chromosomal abnormalities are summarized in S1 Table.

### Whole genome sequencing (WGS) of cell lines SB.06 and SB.07

We performed Whole Genome Sequencing of cell lines SB.06 and SB.07. In both patients, genomic DNA from cell lines was used in parallel with their matched normal DNA (blood in both cases) for WGS. Sequencing was performed by CompleteGenomics using its previously described long fragment read (LFR) technology [7, 9]. Variations between the reference genome and the samples were determined and called by using the previously described CGA Tool^™^ assembly approach [7]. Gross mapping yield (Gb) of all four genomes exceeded 185GB, percentages of fully called bases for tumor and normal of SB.06 and SB.07 exceeded 95.7 percent or greater for both tumor and normal genomes, depth of coverage for fully called variants exceeded 65×, and 100K normalized coverage variability was less than 0.02 in each sample. A detailed summary of quality control (QC) metrics including total numbers and rates of SNPs, insertions, and deletions including ratios of heterozygous versus homozygous and synonymous versus non-synonymous variants is provided in S2 Table.

**Fig 4:**
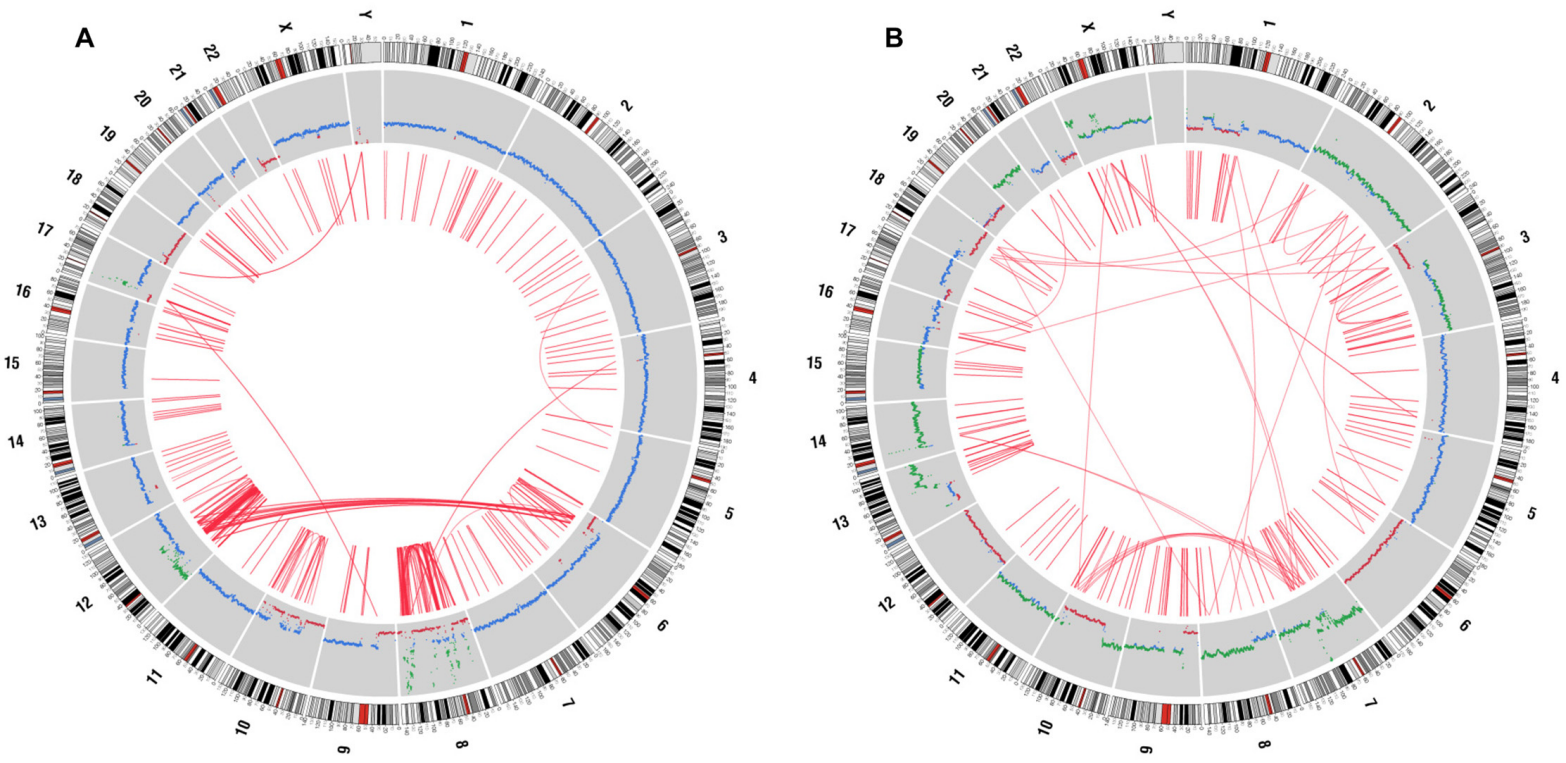
Summary of genomic aberrations in established cell lines. A) SB.06 and B) SB.07 during WGS displayed as Circos plots of both genomes. The outer circle shows copy number variations (red, gains; green, losses); translocations are depicted as links in the interior of the plot.

Chromosomal abnormalities identified by SKY/FISH were compared with results of WGS. Fig 4 shows Circos plots of genomic aberrations for genomes of both SB.06 and SB.07 which was compared to SKY/FISH findings listed in S1 Table: 12 out of 15 and 6 out 10 chromosomal translocations and insertions in cell lines SB.07 and SB.06 were confirmed by WGS, respectively (S1 Table).

### Mutational analysis

To hone in on truly positive somatic mutations, first, any variants which were also found in the matched normal genomic DNA were removed. Secondly, variants previously observed in dbSNP130 or the 1,000 genome project were removed and using previously described somatic variant screening which set true positive and true negative thresholds. A total of 197 and 219 somatic exomic variants were identified in SB.06 and SB.07, respectively (dbGaP; Accession ID phs001042.v1.p1). For SB.06 and SB.07, there were 39 and 50 silent (synonymous) and 142 and 160 protein-altering (non-synonymous) mutations. There were 7 and 8 small deletions and 22 and 19 insertions and 6 and 5 splice region variants. S3 and S4 Tables list all exomic somatic mutations of SB.06 and SB.07, respectively, together with probability analyses on the impact of these mutations on protein structure and function. To validate somatic mutations identified in the coding regions following WGS of SB.06 and SB.07, the results of a previously CLIA-validated 200-gene next generation sequencing assay (Oncovar assay) from cell lines SB.06 and SB.07 were compared to the exonic sequencing results of SB.06 and SB.07 derived from WGS. There was validation of 90 percent of the variants identified by WGS and interrogated by the 200-gene assay (S6 Table).

### Somatic c.2587G>A mutation of plexin A1 receptor exerts gain-of-function of ligand-induced invasion and proliferation

Genes listed in the KEGG database as part of axon guidance signaling (S7 Table) were interrogated for somatic variants present in the WGS results of SB.06 and SB.07: excluding KRAS four out of 158 non-synonymous somatic variants in SB.06, and 2 out of 169 variants in SB.07 involved axon guidance genes (Table 3).

**Table 3:** Somatic mutations in axon guidance genes in pancreatic cancer cell lines SB.07 and SB.07.

Cell line	Gene	Ref	Obs	Mutation
**SB.06**	KRAS	C	G	NM_004985:c.G34C:p.G12R
	PLXNA1	G	A	NM_032242:c.2587G>A:p.D863N
	PLXNA2	C	A	NM_025179:exon5:c.1506+1G>T
	PLXNB2	T	A	NM_012401:c.A3756T:p.E1252D
	ROBO2	-	TCC	NM_002942:c.2294_2295insTCC:p.P765delinsPP
**SB.07**	EPHA8	CCA	AGG	NM_020526:exon7:c.1577_1579AGG
	KRAS	C	A	NM_004985:c.G35T:p.G12V
	PLXNA3	G	C	NM_017514:c.G5209C:p.D1737H

To establish whether somatic mutations affect function of plexin A signaling, the c.2587G>A variant predicted by MutationTaster as disease causing was chosen for further study. S1 and S2 Figs show location of c.2587G>A PLXNA1 D863N variant at the beginning of one of the IPT/TIG (extracellular immune globulin-like fold domains) generated by Protter and the phylogenetic conversation of the residue across eight Euteleostomi [25]. The presence of the mutation was independently validated in both the original SB.06 tumor tissue and the derived SB.06 cell line using a custom-designed Taqman assay (S8 Table). Both, gDNA from the tumor (>50% tumor fraction) and matched normal were isolated by the study pathologist (K.J.K.) and together with gDNA of Panc1 cells as negative control subject to mutation testing. Mutant PLXNA1 c.2587G>A was exclusively detected in SB.06 tumor tissue and SB.06 cells but not in SB.06 uninvolved pancreas or Panc1 cells (S8 Table).

**Fig 5:**
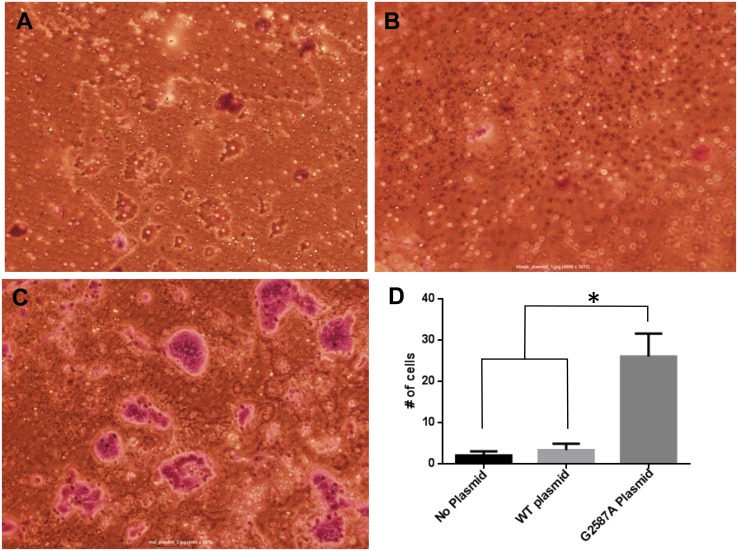
Mutant PLXNA1 mediates invasion of 293T following transfection. Invasion of 293T cells transfected with A) no plasmid, B) WT plasmid and C) mutated PLXNA1 c.2587G>A plasmid, 48 hours after administration of 150 ng/mL semaphorin 3A. *p< 0.05 via 2-tailed Student’s t-test.

Both, wild type (WT) and c.2587G>A mutant PLXNA1 cDNA made via site-directed mutagenesis was cloned into mammalian expression vectors and in addition to empty (mock) vector expressed in 293T and Panc1 cells. Both transcription of the recombinant gene with the G to A substitution at position 2587 was observed at the mRNA level by Sanger sequencing of cDNA, and equal expression of WT and mutant PLXNA1 expression was confirmed by Western Blotting against the HA-Flag epitope present on the carboxy terminus of the recombinant PLXNA1 proteins upon transfection of 293T and Panc1 cells. Ligand-induced invasion of 293T cells was exclusively observed in cells transfected with c.2587G>A mutant PLXNA1 and not in wild type PLXNA1-transfected cells (Fig 5).

**Fig 6:**
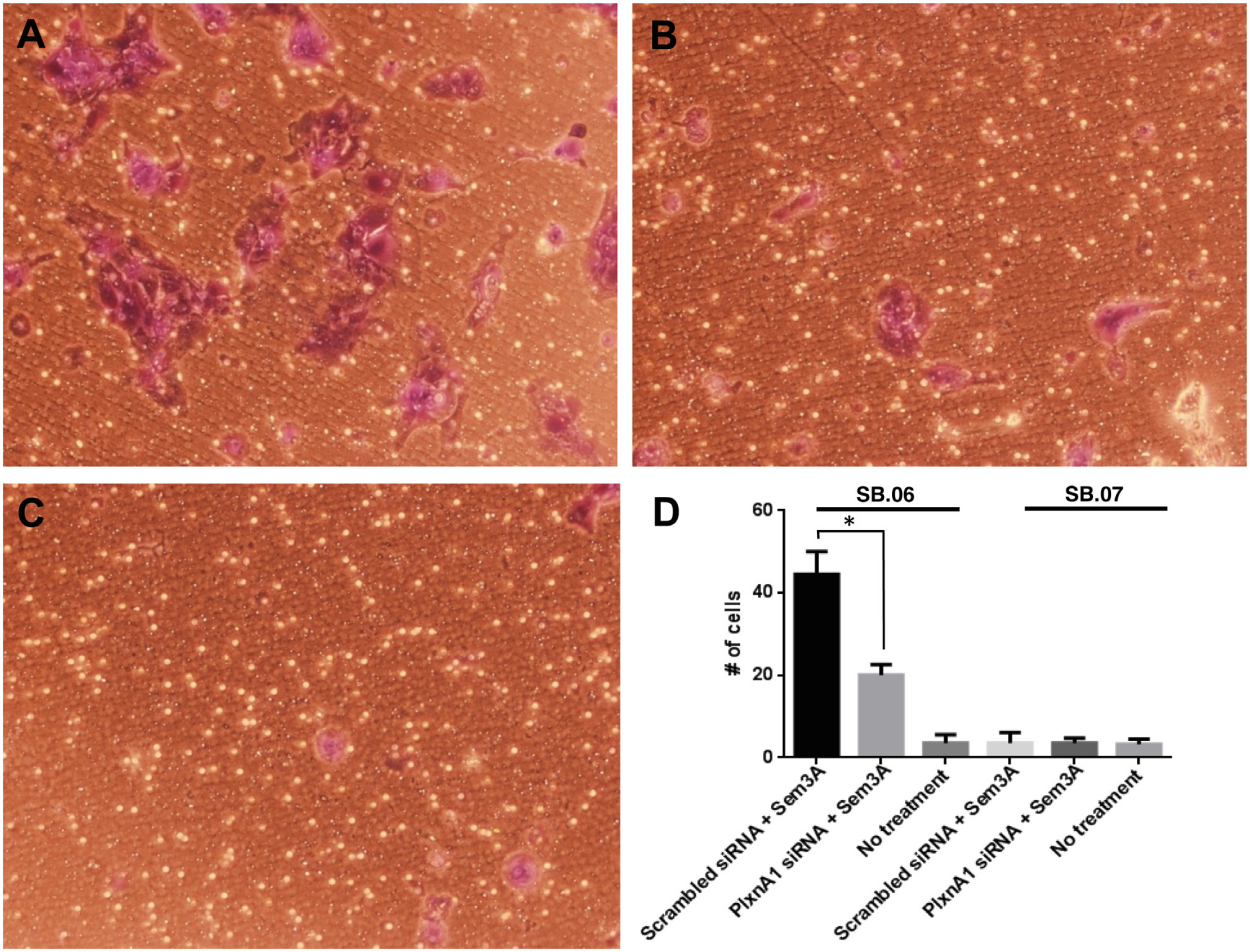
Mutant PLXNA1 mediates ligand-induced invasion of SB.06 cells. A) Invasion of SB.06 cells treated with scrambled siRNA and Sem3A(150 ng/mL), B) PLXNA1 siRNA knockdown and Sem3A, and C) intact SB.06 cells with no treatment. D) Quantification of SB.06 invasion compared with equivalently treated SB.07 cells. * p<0.05 via 2-tailed Student’s t-test.

**Fig 7:**
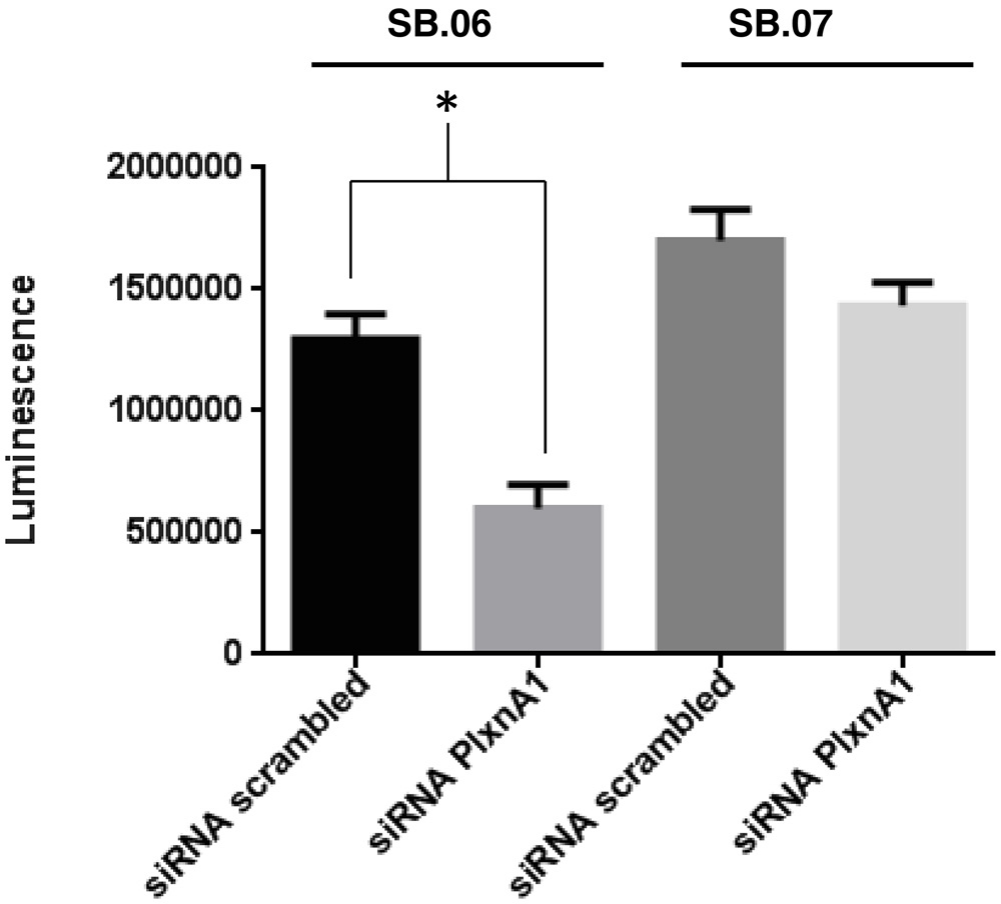
Proliferation of SB.06 cells harboring mutant PLXNA1 is reduced following RNAi silencing of PLXNA1. Cell growth of SB.06 and SB.07 cell lines following siRNA knockdown of PLXNA1 and 48 hours of treatment with 150 ng/mL of Sem3A, as measured by Cell-Titer Glo assay. *p<0.05 via 2-tailed Student’s t-test.

**Fig 8:**
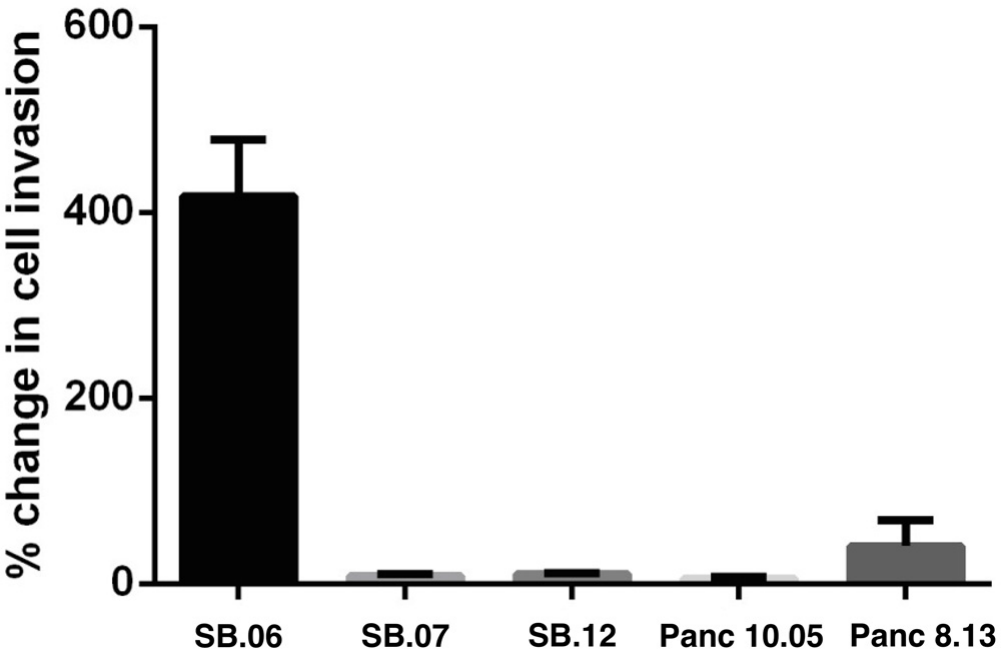
Invasion of various pancreas cancer cell lines in response to semaphorin stimulation. SEMA3A stimulation (150ng/mL) increases invasion of SB.06 cells, but not of pancreas cell lines not harboring a PLXNA1 mutation.

To evaluate a possible selective gain-of-function of mutant PLXNA1 signaling in pancreas cancer, PLXNA1 was silenced in both SB.06 and SB.07 cells via introduction of PLXNA1 siRNA. Invasion through Matrigel chambers was observed in SB.06 cells when co-incubated with 150 ng/mL of SEMA3A ligand (Fig 6A). Upon PLXNA1 siRNA knockdown there was a 50% drop in the number of SB.06 cells invading through the Matrigel (Fig 6D). Proliferation was selectively reduced in SB.06 cells upon knockdown of the mutated PLXNA1 but not in SB.07 cells which do not harbor PLXNA1 mutations (Fig 7). Treatment with 150ng/ml of PLXNA1 receptor ligand SEMA3A induced invasion only in SB.06 but not in SB.07, SB.12, Panc10.05, and Panc08.13 cells (Fig 8), which do not harbor PLXNA1 mutations.

### Mutant PLXNA1 signaling implies Rho-GTPase CDC42 as downstream effector

Considering the involvement of plexin receptor signaling in cytoskeletal organization, cell polarity, and growth cytoskeletal Rho GTPase signaling pathways including effectors of RhoA-GTPase, Rac1, and CDC42 were examined in wild type and c.2587G>A PLXNA1 mutant expressing 293T and Panc1 cells using gene expression profiling and phospho-immunoblotting. When comparing genes involved in Rho-GTPase signaling pathways using the Rho pathway RT^2^ Profiler^™^ PCR Array ROCK1 and SEPT9 were exclusively expressed in 293T cells transfected with c.2587G>A mutant PLXNA1 and not with wild type PLXNA1. Expression level of several other Rho effectors were selectively increased in mutant c.2587G>A PLXNA1 expressing cells compared to WT cells (Table 4).

**Table 4:** Gene expression changes measured by Rho pathway qPCR array in 293T cells transfected with PLXNA1 plasmid.

Gene	WT expression	Mutant expression	Fold upregulation in mutant
***SEPT9***	-	+	N/A
***ROCK1***	-	+	N/A
***RELA***	+	+	3.28
***PIK3CA***	+	+	8.14
***PRKCI***	+	+	3.64
***EZR***	+	+	7.33
***IL8***	+	+	3.26
***JUN***	+	+	4.94
***CDC42EP2***	+	+	2.58

**Fig 9:**
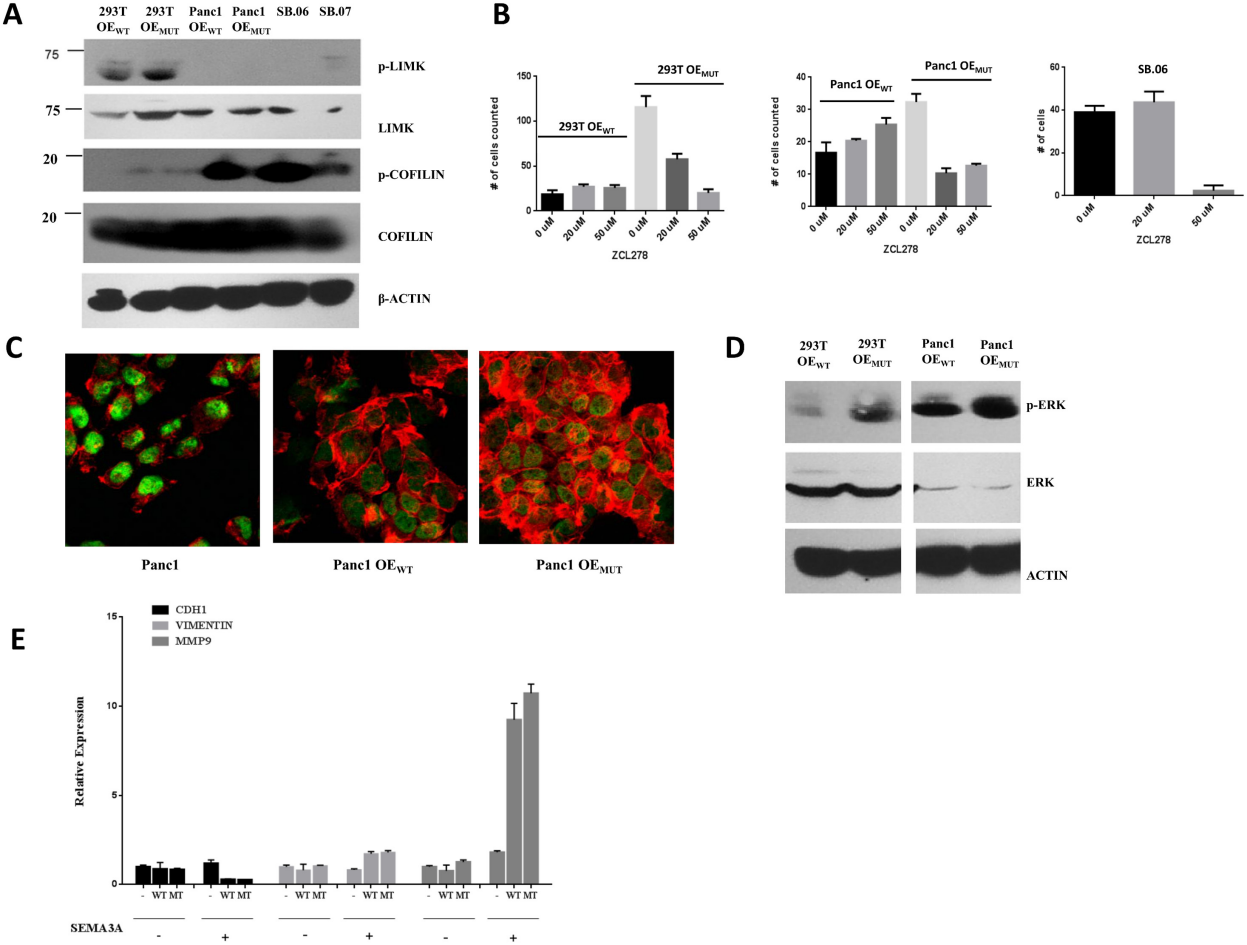
Mutant c.2587G>A PLXNA1 activates RhoGTPase and p44/p42 MAPK signaling. A) Increased activation of RhoGTPase signaling effectors cofilin and LIMK in 293T and pancreas cancer cells expressing mutant c.2587G>A PLXNA1 (293T OE_WT_, 293T cells overexpressing wild type PLXNA1; 293T OE_MUT_, 293T cells overexpressing mutant); B) Pharmacological inhibition with the CDC42-specific small molecule inhibitor ZCL278 abrogates invasion and migration in 293T and Panc1 cells overexpressing mutant c.2587G>A, but not wild type PLXNA1, as well as SB.06 cells harboring mutant c.2587G>A PLXNA1; C) Increased actin filaments and cytoplasmatic expansion in Panc1 cells transfected with mutant PLXNA1 versus PLXNA1 wild type; D) Overexpression of c.2587G>A PLXNA1 increases p-Erk levels in 293T and pancreas cancer cells compared to wild type PLXNA1. Immunoblot of 293T and Panc1 cells probed with Erk and actin antibodies; E) Expression of the epithelial-to-mesenchymal transition markers E-cadherin, vimentin, and metalloproteinase MMP-9 analyzed by qRT-PCR (normalized ΔCt values are shown). 293T cells overexpressing wild type PLXNA1 or mutant (MT) c.2587G>A PLXNA1 were treated with 150ng/ml SEMA3A (+) or vehicle (-) for 10 minutes prior to RNA harvest and qRT-PCR analysis.

To confirm altered Rho-GTPase signaling in mutant PLXNA1 cells, 293T and Panc1 cells transfected with wild type or mutant PLXNA1, or SB.06 harboring mutant and SB.07 cells harboring wild type endogenous PLXNA1 levels were probed with anti-phospho antibodies against the Rho-GTPase effectors and cytoskeletal and AMP/actin regulators LIM kinase (LIMK) and cofilin. Fig 9A shows phospho-cofilin levels >10-fold elevated in Panc1 cells transfected with PLXNA1 c.2587G>A mutant compared to wild type PLXNA1, and increased in 293T PLXNA1 c.2587G>A compared to 293T cells expressing wild type PLXNA1. p-cofilin levels were also increased in SB.06 compared SB.07 cells. LIMK levels were elevated in 293T cells transfected with mutant compared to cells transfected wild type PLXNA1 receptor. To show that increased Rho-GTPase signaling mediates, in part, the observed increase in invasion and migration cells expressing wild type and mutant PLXNA1 were treated with the CDC42-selective small molecule inhibitor ZCL278. The molecule selectively targets the binding site of the CDC42 guanine nucleotide exchange factor intersectin (ITSN) and has previously been shown to inhibit a number of CDC42-mediated cellular effects [26]. Fig 9B shows selective suppression of increased migration in both 293T and Panc1 cells transfected by mutant PLXNA1 after administration of the CDC42 inhibitor with levels of invasion in mutant LXNA1 cells returning to levels of wild type PLXNA1 cells and wild type PLXNA1 expressing cells not changing invasion upon treatment. Equally, treatment with the anti-CDC42 small molecule inhibitor suppressed invasion and migration in the SB.06 cells where the mutation was identified (Fig 9B).

Cofilin belongs to a family of related proteins called the actin depolymerizing factor (ADF)/cofilin family, inhibiting polymerization of actin filaments. Depending on the kinetics and turnover of actin, in cancer cells decreased levels of polymerized, condensed actin filaments can be seen as an ‘expanded’, less polarized cytoplasm due to an increase in non-polymerized, untangled actin filaments. To confirm that the different activation state of cofilin in Panc1 cells transfected with wild type versus mutant PLXNA1 impairs cytoskeletal organization, cytoskeletal morphology was measured by confocal microscopy using anti-phalloidin as a high-affinity filamentous actin (F-actin) probe. Fig 9C shows increase of actin filaments and cytoplasmic expansion in Panc1 cells transfected with mutant PLXNA1 versus wild type. Probing for additional mechanisms of increase in invasion mutant c.2587G>A PLXNA1 was also associated with increased pERK levels (Fig 9D). While overexpression of PLXNA1 in 293T cells governed different responses to SEMA3A stimulation, expression levels of E-cadherin, MMP-9, and vimentin did not differ between wild type and mutant PLXNA1-transfected 293T cells (Fig 9E).

To examine if PLXNA1 mutation status might aid to guide molecular therapy decisions, a panel of primary pancreas cancer cell lines with known PLXNA1 mutation status including SB.06 (PLXNA1 c.2587G>A), SB.07, Panc01.28. Panc06.05, Panc08.13, and Panc10.05 (all wild type) were treated with the EGFR inhibitor erlotinib and the dual PI3K/mTOR inhibitor BEZ235 (S3 Fig). While the inherent genetic heterogeneity across the tested primary pancreatic cancer cell lines opens alternate explanations for any observed drug-phenotype correlations, we hypothesized that receptor tyrosine kinase-independent, genetically driven alternate activation of the MAPK pathway, like through mutant PLXNA1 signaling associated with elevated pErk levels (Fig 9D), might display a distinct drug phenotype. SB.06 cells showed increased resistance to both anti-EGFR and anti-PI3K inhibition compared to SB.07 and other tested pancreas cancer lines as measured by ≥3 to 10-fold higher IC50 values in treated SB.06 cells compared to other lines (S3 Fig). These results may indicate that dysregulated downstream signaling of PLXNA due to somatic variants affecting the PLXNA1 receptor leading to elevated pERK levels might mediate resistance cues to molecular therapy approaches of pancreas cancer.

## Discussion

Patient-derived well-characterized cancer cell lines have proved an invaluable resource for research efforts in pancreas cancer. Studies carried out in cancer cell lines which were pivotal for recent progress and which harbored translational significance included, as examples, work on aberrant immunological signaling, genomic stability, or drug phenotype-genotype correlations [20, 27–29]. Using two previously described methods, we established an additional three lines whose identity was confirmed by a combination of immunophenotyping, HLA genotyping, and karyotyping [3, 20]. We aimed to identify novel drivers of pancreas cancer progression by WGS and to evaluate variants on cancer biology, which might inform in future drug development. Pancreatic cancers harbor a lower number of somatic mutations per tumor compared to many other solid organ malignancies [30]. The number of protein-altering genetic variants detected through sequencing and copy number analyses in the first comprehensive study on the genomic landscape of pancreas cancer performed on patient-derived xenotransplants and in vitro cell lines was reported as 62 per tumor with nearly two-thirds due to missense mutations and the other third due to deletions, translocations, and amplifications [17]. Using WGS on patient tumors a follow-up study reported the average number of mutations detected per patient was 26 (range 1–116) [9]. The inter-tumoral heterogeneity of somatic mutations between pancreatic cancers is relatively high. With the exception of the dominant, near omnipresent activating Kras mutations and the three tumor suppressor variants in CDKN2A (p16), SMAD4, and TP53, other recurrent mutations involving the same gene are rare [31]. Variants affecting TGFBR2, ATM, MLL6, MAP2K4, NALCN, SLC16A4 and MAGEA6 genes have been measured with a frequency of less than 10 percent [9, 31]. These and the majority of non-recurring low frequency mutations cluster on at least 11 core-signaling pathways, and pathways affected by somatic variants show a high inter-tumoral variability [17, 32]. While these genetic screens are sentinel studies on the genetic landscape of pancreas cancer, the great majority of identified mutations did not undergo functional validation so far.

When comparing the mutation profile of cell line SB.06 with SB.07 we noticed a greater number of mutations affecting axon guidance (AG) genes in SB.06 cells. This observation together with the lack of a bona fide in vitro validation of previously identified mutations in AG genes prompted us to study the role of these mutations in greater detail. We selected the PLXNA1 c.2587G>A variant for further study for a number of reasons: Gene expression studies have recently implied plexin A receptor–SEMA3A signaling in pancreas cancer progression [9, 12]. Patients with high transcript levels of the ligand or the PLXNA1 receptor were experiencing shorter overall survival. Such a suggested gain-of-function was also reported in a smaller correlative tissue study by Mueller *et*. *al*. showing transcript levels of PLXNA1, SEMA3A, and the neuropilin-1 co-receptor required for SEMA3A-mediated plexin A receptor activation upregulated in stage-specific manner [12]. High PLXNA1 and SEMA3A levels were associated with adverse outcome. We also selected plexin A1 receptor mutations found in SB.06 for further study due to the unusual growth pattern of these cells which display formation of swirls at subconfluency. As axon guidance signaling transmits both autocrine and paracrine signals governing cytokinesis of cells via expansive and repulsive signals we speculated that dysregulated AG gene signaling in SB.06 cells due to the multiple genetic perturbations of AG genes, involving both the semaphorin–plexin A and SLIT ligand-ROBO receptor axes in these cells, might be linked to the observed unusual growth pattern which resembles a “rosette” formation picture known from histopathological sections of neuroendocrine carcinoid tumors [33, 34].

Semaphorin 3A–plexin A1 receptor signaling predominantly mediates repulsive signals with regard to cytokinesis and cell growth inducing collapse of actin polymerization and clusters and budding cell cones [11, 12]. However, studies of PLXNA1 –SEMA3 signaling in glioblastoma and pancreas cancer have pointed to an opposite role of promotion of migration, invasion, and growth presumably through different regulation and function of effector downstream signaling. Semaphorins 3 activates plexin receptors in both a neuropilin-dependent and independent way. SEMA3A, as well as SEMA3B-D, activate plexin A1 signaling via neuropilins (NPN) whereas SEMA6D, E activates PLXNA1 via direct binding [33]. SEMA3A binding to NPN-1 of the NPN-1–PLXNA1–SEMA3A holocomplex induces a conformational change in the complex which dissociates the plexin A1-SEMA3A complex [35]. The plexin A1–SEMA3A complex binds both via SEMA3A and the remainder of the ectodomain of the plexin A1 receptor to different sites of NPN-1. Release of the plexin A1–SEMA3A interaction via the conformational change induced by SEMA3A binding to NPN-1 creates a new homeostasis of plexin A1–NPN-1 protein-protein interactions. This release of SEMA3A-triggered autoinhibition generates a number of downstream changes. One of the best understood downstream signaling cascades of activated PLXNA1 includes the activation of the adaptor protein Ras GTPase activating protein (RasGAP) which is bound to the intracytoplasmic domain of the PLXNA1 receptor, starting with stimulation of intracytoplasmic plexin A RasGAP activity, by the release of the Rac guanide exchange factor (GEF) adaptor protein ‘FERM, RhoGEF and pleckstrin domain protein 2’ (FARP2) leading to Rac1 and CDC42-PAK activation. In light of the altered response to the CDC42 inhibitor ZCL278, it is tempting to speculate that c.2587G>A mutations in the PLXNA1 receptor preferentially affect signal transduction through this pathway whereas the down regulation of E-cadherin and increase in MMP9 expression upon SEMA3A stimulation, which showed no difference in wild type versus mutant PLXNA1 overexpressing cells, is mediated through different mechanisms by other domains of the molecule. Additionally, our finding that upon stimulation with the PLXNA1-ligand SEMA3A RNAi silencing of the PLXNA1 receptor reduces only half of the induced invasion and reduces proliferation only in mutant, but not wild type, PLXNA1 expressing cells together with mutant PLXNA1 overexpression strongly activating cofilin signaling but not altering MMP9 or E-cadherin expression upon SEMA3A treatment further supports the known pleotropism and complexity of SEMA3A ligand signaling. For example, different domains responsible for homo- and heterodimer formation of PLXNA1 receptors guiding select downstream signaling have recently been identified, and a recent report from prostate cancer links upregulation of PLXNA2, also a receptor for the ligand SEMA3A, but not upregulation of MMP9, to induced PC3 cell migration and invasion [36, 37]. Cofilin activation was not measured in this study [37].

The plexin A1 receptor mutation c.2587G>A induces an amino acid change from aspartic acid to asparagines at position 863 substituting an amino acid residue with a negative side-chain charge with an amino acid with a neutral one in the third ‘plexin, semaphorin, and integrin domain’ (PSI) of the plexin receptor (S1 Fig). PSI domains are cysteine-rich modules found in extracellular fragments of hundreds of signaling proteins, including plexins, semaphorins, integrins, and attractins. They are located in plexin receptors between the N-terminal SEMA binding domain and the four “Ig-like, plexins, transcription factors” (IPT) domains which are found in many cell surface receptors including MET and RON as well as in intracellular transcription factors. The PSI, and IPT, domains are likely involved in protein-protein interactions of the plexin A1 receptor with other membrane receptors including NPN-1, other plexin receptors, or proto-oncogenes like MET and RON which harbor PSI and IPT domains. The induced amino acid changes in a highly conserved region with the introduction of a more positively charged milieu into the plexin A1 structure through the D863N switch suggests, based on its biochemical affinity alterations as measured by, for example, the SIFT score, altered protein function of the variant PLXNA1 receptor (S4 Table). Mutations affecting these critical regulatory regions of plexin receptors have previously been described in melanoma and pancreas cancer and the enrichment of mutations affecting these regulatory regions supports a possible role in mediation of cues promoting growth and survival possibly even opening avenues for therapeutic interventions as discussed by some authors [15, 38]. It is conceivable that altered protein-protein interactions either via different folding of the PSI domain or through increased direct affinity to potential binding partners lock the receptor into a state of constitutive activation and altered signal transduction. Alternatively, the abrogation of some but not all of the increased invasion induced by SEMA3A in PLXNA-mutant cells might be due to the effects of SEMA3A onto other plexin receptors like plexin A2 and A3, and thus due to any induced misbalances of ligand and receptor homeostasis in the finely regulated network. For example, plexin A3 receptor expression shows a different gene expression profile in normal pancreas and pancreas cancer biospecimens and across pancreatic cancer lines suggesting different functions. Data from other cancers suggest the involvement of PLXNA2 and PLXNA3 in cell extension and invasion [37, 39]. Disturbances of the finely-tuned cytoskeletal organization and cytokinesis governed by plexin–semaphorin signaling and other regulators of the axon gene family have previously been shown to foster a malignant evasive phenotype, and it is conceivable that altered plexin receptor A1 function through somatic mutations like D863N tips cell-extending and invading signaling cues by SEMA3A-stimulated plexin signaling towards cell extension, invasion, and growth in pancreatic cancer [40–42]. Of note, PLXNA2 signaling, found to be affected by somatic mutations in SB.06 cells, has also been found to be associated with adverse outcome in previous studies in pancreas cancer, as well as with a pro-invasive phenotype in prostate cancer. It can be speculated that both PLXNA1 and A2 receptors might need to be silenced for SEMA3A-induced invasion levels in PLXNA-mutant SB.06 cells to return to baseline levels. The presence of somatic variants in both PLXNA1 and A2 receptors might also be needed to cause, or contribute to, the swirl-like growth pattern of these cells.

It is intriguing that gene expression findings obtained from 293T cells transfected with wild type or mutant PLXNA1 align, in part, with previous findings of activated downstream signaling of plexin A1 receptors in pancreas cancer cells. Plexin A1 activation via SEMA3 treatment in different pancreas cancer cell lines has been previously shown to activate Rac1, GSK3β and p44/p42 MAPK signaling. As one of its suggested intracellular signaling mechanisms, it has been shown that following SEMA3A stimulation, FARP2 (a RacGEF) dissociates from the cytoplasmic tail of the PLXNA1 receptor. FARP2 then activates Rho-GTPases including Rac1 which can activate NF-κB subunit RelA, Jun, and CDCc42. This mechanism is in line with the observed gene expression changes of the Rho Pathway PCR array and the other downstream findings of mutant PLXNA1 expressing cells: the upregulation of the CDC42 effector protein 2 (CDC42EP2) and the downstream regulators of cytoskeletal organization like EZR and PRKCI, the observation that the CDC42-selective small molecule inhibitor returns elevated immigration and invasion in 293T and pancreas cancer harboring mutant PLXNA1 to baseline, and the increased activation of the CDC42-downstream effectors cofilin and LIMK combined with concomitant morphological changes of cytoskeletal disorganization and expansion suggests activation of the Rho-GTPase CDC42 as one of the mediators of mutated PLXNA1 signaling. That somatic mutations in the PLXNA1 receptor might exert similar downstream effects usually as seen after ligand stimulation is also supported by the observation of increased phospho-Erk levels and unchanged E-cadherin and MMP-9 levels between SEMA3A-stimulated and untreated pancreas cancer cells including Panc1 described in a prior report [12]. Muller and coworkers reported previously that SEMA3A treatment of pancreas cancer cells increases phospho-Erk levels but not expression levels of MMP2, MMP9, or markers of EMT, and suggest that these are, in a ‘pancreas cancer-specific character’ not a part of SEMA3A downstream signaling in pancreatic cancer cells [12]. Since c.2587G>A mutant PLXNA1 in our study similarily increases pErk levels, but not MMP9 or markers of EMT, we would like to offer the observed SEMA3A-induced cytoskeletal re-organization and dysfunction via CDC42-mediated cofilin activation as possible PLXNA1 downstream signaling and mediator of increased migration in pancreas cancer.

In light of elevated p-ERK levels in mutant PLXNA1 overexpressing cells, we subjected both SB.06 and SB.07 cells to treatment with the EGFR inhibitor erlotinib and the dual PI3K/mTOR inhibitor BEZ235 and measured an increased resistance in SB.06 cells compared to SB.07 and other wild type PLXNA1 harboring pancreas cancer cells. If this drug phenotype is directly linked to dysregulated plexin A1 receptor signaling due to the somatic mutations affecting PLXNA1, and possibly A2, genes and increased PI3K activity is due to the multiple confounding factors of the impact of additional mutations currently unknown.

In summary, we have identified novel axon guidance mutations in newly established pancreas cancer lines within a whole genome sequencing effort. We evaluated one of these mutations affecting the plexin A1 receptor and show a bona fide enhancing impact on ligand-induced invasion and proliferation in cell-based models. Comparative functional studies on cells transfected with mutant and wild type plexin receptor A1 provide leads for aberrant effector function of mutant PLXNA1 signaling which might provide leads for possible future molecular genotype-directed therapy approaches. Identification followed by functional characterization of low frequency somatic variants in pancreas cancer may have translational value for improved future personalized treatment efforts in a deadly disease.

## Supporting Information

S1 Fig Schematic diagram showing the location of *c*.*2587G>A PLXNA1* D863N variant: magnified start of the IPT/TIG (extracellular immune globulin-like fold domains) generated by Protter is shown. (TIF)

S2 Fig The phylogenetic conversation of the residue across eight Euteleostomi. (TIF)

S3 Fig Dose response curves for erlotinib and BEZ-235 of various cell lines: SB.06 (red), SB.07 (green), Panc 10.05 (blue), Panc08.13 (yellow), Panc06.05 (purple) and Panc01.28 (orange) treated with A) erlotinib and B) BEZ-235, representative of three independent experiments performed in triplicate. (TIF)

S1 Table Chromosomal abnormalities identified in established cell lines SB.06, SB.07, and SB.12. (XLSX)

S2 Table Summary of whole genome sequencing metrics of SB.06 and SB.07. (XLSX)

S3 Table Summary of exomic somatic variants identified in SB.06 by whole genome sequencing; see S5 Table for annotation legend. (XLSX)

S4 Table Summary of exomic somatic variants identified in SB.07 by whole genome sequencing, see S5 Table for annotation legend. (XLSX)

S5 Table Annotation legend for ANNOVAR whole genome sequencing tables. (XLSX)

S6 Table Detected variants for both whole genome sequencing (WGS) and Oncovar assay in cell lines SB.06 and SB.07. (XLSX)

S7 Table Symbols and names of genes in axon guidance pathway. (XLSX)

S8 Table PLXNA1 mutation status in SB.06 cells and tissues. (XLSX)

S9 Table Symbols and names of genes included in Oncovar assay. (XLSX)
