# Supplementary figures and images for: Whole Genome Sequencing of Newly Established Pancreatic Cancer Lines Identifies Novel Somatic Mutation (c.2587G>A) in Axon Guidance Receptor Plexin A1 as Enhancer of Proliferation and Invasion
Source: PLoS One. 2016 Mar 10;11(3):e0149833. doi: 10.1371/journal.pone.0149833 (PMC4786220; doi:10.1371/journal.pone.0149833)

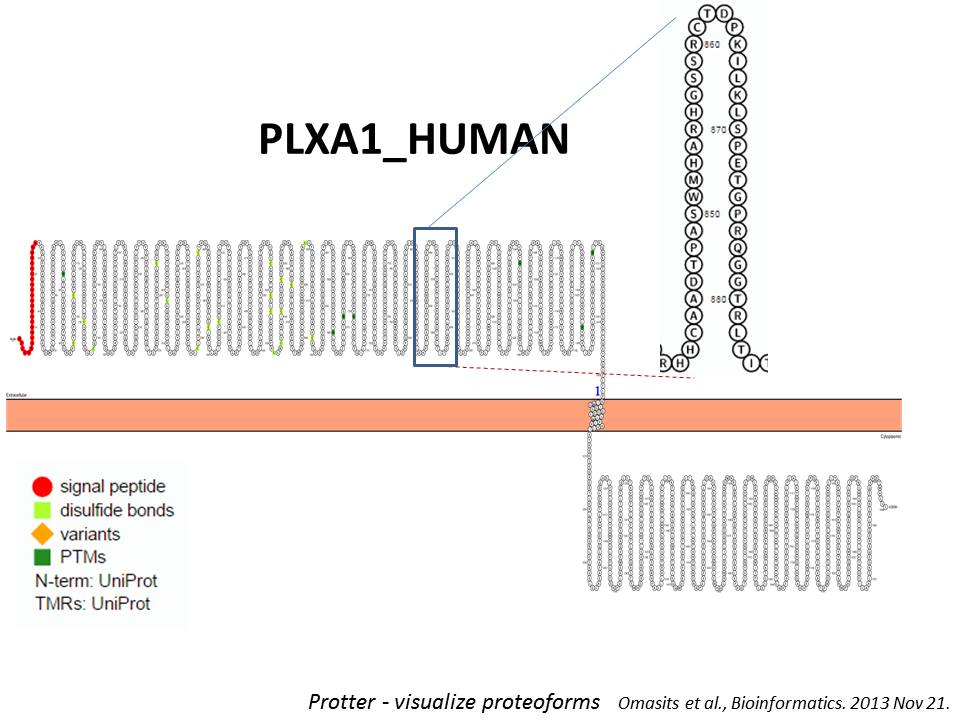

Supplement: S1 Fig — (TIF) [file pone.0149833.s001.tif]

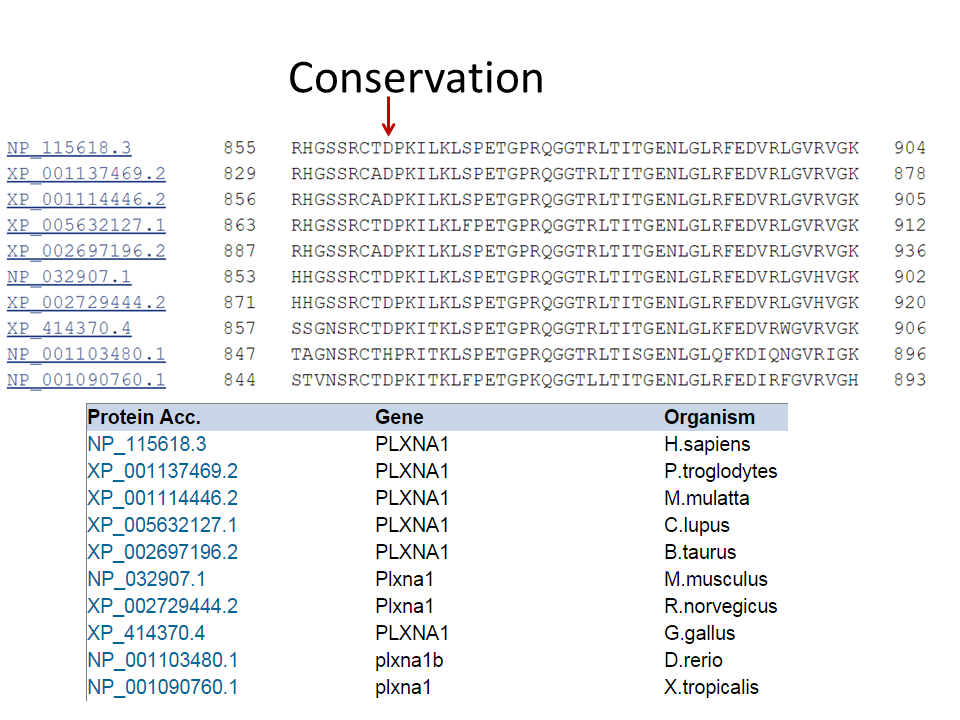

Supplement: S2 Fig — (TIF) [file pone.0149833.s002.tif]

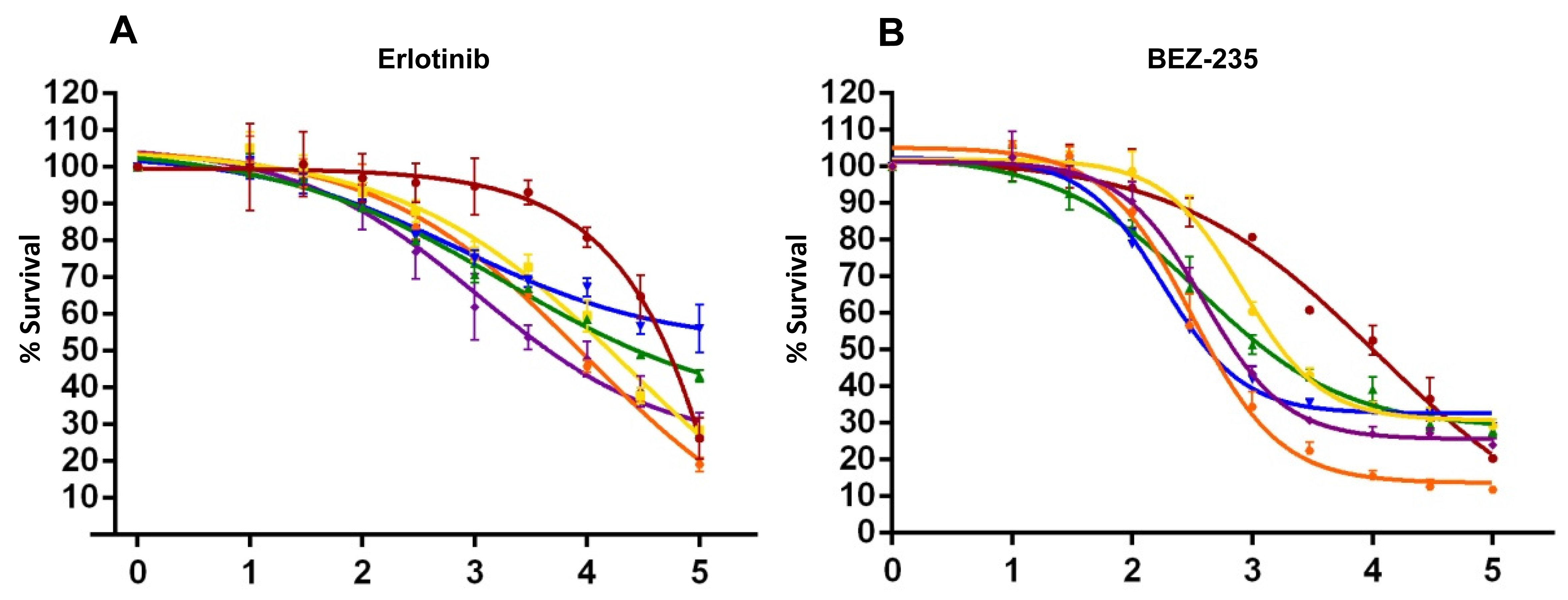

Supplement: S3 Fig — (TIF) [file pone.0149833.s003.tif]
